# Quality control in veterinary blood banks: evaluation of canine platelet concentrates stored for five days

**DOI:** 10.1186/s12917-020-2254-5

**Published:** 2020-01-30

**Authors:** Camila Serina Lasta, Nicole Hlavac, Natália Aydos Marcondes, Magnus Larruscaim Dalmolin, Silvia Resende Terra, Luciana de Almeida Lacerda, Gustavo Adolpho Moreira Faulhaber, Félix Hilário Díaz González

**Affiliations:** 1 Departamento de Saúde, Faculdade de Medicina Veterinária, Centro Universitário Ritter dos Reis – UniRitter – Campus FAPA, Av. Manoel Elias, 2001, 91240-261, Porto Alegre, Brazil; 2 Departamento de Patologia Clínica Veterinária, Universidade Federal do Rio Grande do Sul, Porto Alegre, Brazil; 3 Departamento de Anatomia, Patologia e Clínicas Veterinárias, Universidade Federal da Bahia, Salvador, Brazil; 4 Laboratório Zanol, Porto Alegre, Brazil; 5 Blut’s Centro de Diagnósticos Veterinários, Porto Alegre, Brazil; 6 Departamento de Medicina Interna, Universidade Federal do Rio Grande do Sul, Porto Alegre, Brazil; 7 Programa de Pós-Graduação em Medicina, Ciências Médicas, Universidade Federal do Rio Grande do Sul, Porto Alegre, Brazil

**Keywords:** Dog, Platelet concentrate, Platelet-rich plasma, Platelet storage lesions

## Abstract

**Background:**

Platelets undergo structural, biochemical and functional alterations when stored, and platelet storage lesions reduce platelet function and half-life after transfusion. The objective of this study was to evaluate stored canine platelet concentrates with platelet aggregation, flow cytometry and biochemistry assays. Twenty-two bags of canine platelet concentrates were obtained by the platelet-rich plasma method and were assessed on days 1, 3 and 5 after collection. Parameters such as platelet counts, residual leukocytes, platelet swirling, glucose, lactate, pH, CD62P expression (platelet activation), JC-1 (mitochondrial function) and annexin V (apoptosis and cell death) were assessed.

**Results:**

Over the five days of storage there was a significant decrease in glucose, HCO_3_, pCO_2_, ATP, pH, swirling and mitochondrial function, associated with a significant increase in lactate levels and pO_2_. At the end of storage pH was 5.9 ± 0.6 and lactate levels were 2.8 ± 1.2 mmol/L. Results of the quality parameters evaluated were similar to those reported in human platelets studies. The deleterious effects of storage were more pronounced in bags with higher platelet counts (> 7.49 × 10^10^/unit), suggesting that canine platelet concentrates should not contain an excessive number of platelets.

**Conclusions:**

Quality parameters of canine platelets under standard storage conditions were similar to those observed in human platelets. Our results have potential to be used for the routine evaluation and quality control in veterinary blood banks.

## Background

Recent advances in intensive care and oncology have increased the demand for blood components in veterinary medicine. However, there is limited availability of platelet concentrate (PC) because it is difficult to obtain and has a very short storage period. Although some studies evaluating the quality of canine PC obtained by the platelet-rich plasma (PRP) method under routine blood bank processing and storage have been reported [1–4], the quality control in veterinary transfusion medicine is still based on human blood bank protocols.

During storage platelets undergo structural, biochemical and functional changes, also referred as platelet storage lesions (PSL), which are caused by a multifactorial process that includes energy consumption, pH decrease, platelet activation and apoptosis [5, 6]. PSL reduce platelet half-life and function after transfusion, accordingly, one of the objectives of veterinary transfusion medicine is to understand and minimize them [7–10]. Consequently, understanding the mechanisms of canine PSL may serve as a basis for future research aimed at developing strategies to increase platelet viability. The objective of our study was to evaluate the main platelet quality parameters in stored canine PC under standard storage conditions.

## Results

**Fig. 1:**
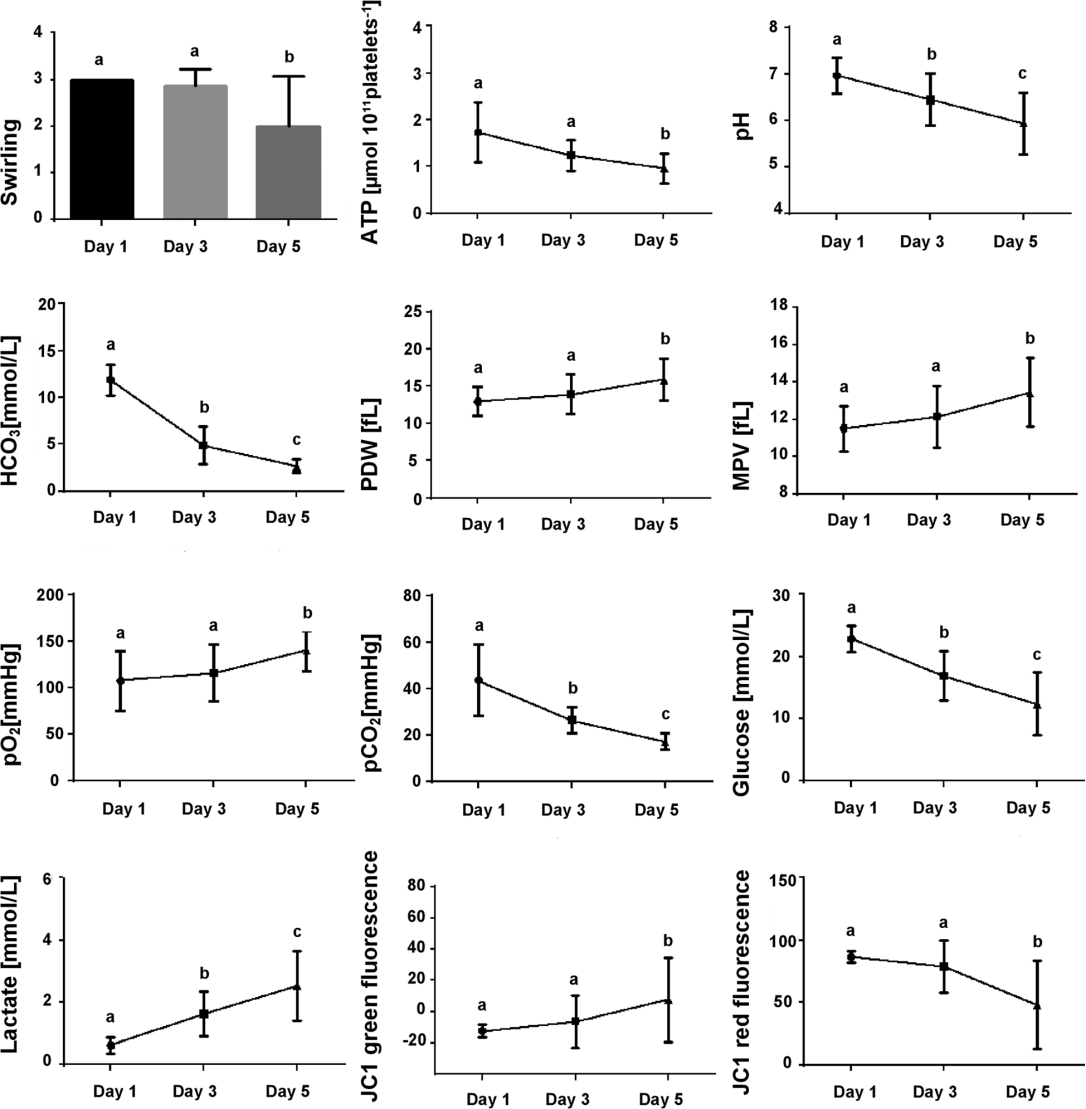
In vitro parameters of canine platelet concentrates stored for five days. Superscript letters represent statistically significant differences among evaluated days (*p* < 0.05)

Data on the different parameters analyzed on days 1, 3, and 5 of storage are showed in Table 1 and Fig. 1. Mean platelet count was 7.48 ± 2.39 × 10^10^/unit on day 1, and it remained stable over time. All PC showed adequate platelet counts, pH, swirling and leukoreduction (residual leukocytes < 2.0 × 10^8^/unit) (data not shown) at the first day of assessment, in compliance with the Brazilian regulations for human PC [11]. Swirling decreased in all PC units over time (*p* < 0.001). None of the canine PC units presented aerobic or anaerobic microbiological contamination.

**Table 1:** In vitro metabolism of canine platelets stored for 5 days (*n* = 22)

Parameters	Day 1	Day 3	Day 5
Platelet count (× 10^10^/unit)	7.48 ± 2.39	7.69 ± 2.39	7.42 ± 2.79
MPV (fL)	11.5 ± 1.2	12.5 ± 4.5	13.2 ± 6.6*
PDW (fL)	13.0 ± 7.9	14.0 ± 5.5	15.1 ± 2.0*
pH (22 °C)	7.0 ± 0.4	6.4 ± 0.5*	5.9 ± 0.7**
pO_2_ (mmHg)	107.6 ± 32.2	115.9 ± 31.0	140.1 ± 21.8*
pCO_2_ (mmHg)	43.7 ± 3.1	25.5 ± 2.5**	9.5 ± 2.5**
Glucose (mmol/L)	25.3 ± 2.3	18.8 ± 4.4**	13.7 ± 5.6**
HCO_3_ (mmol/L)	11.9 ± 1.7	3.5 ± 2.8**	0.6 ± 1.2**
Lactate (mmol/L)	0.7 ± 0.3	1.8 ± 0.8*	2.8 ± 1.2**
ATP (μmol/10^11^platelets)	1.7 ± 0.6	1.2 ± 0.3*	1.0 ± 0.3**
CD61 (%)	91.5 ± 4.8	93.4 ± 4.3	91.8 ± 5.7
CD62P (%)	2.7 ± 1.5	2.6 ± 3.6	2.6 ± 2.2
LDH (U/L)	237.9 ± 159.1	404.7 ± 98.5	630.0 ± 162.1
Annexin V (%)	2.4 ± 2.5	1.6 ± 1.1	3.5 ± 4.2
Caspase (%)	10.2 ± 11.4	6.7 ± 5.9	12.0 ± 13.9
∆Ψm JC-1 (%)	91.8 ± 3.9	84.2 ± 20.5	55.4 ± 37.3**

Data are shown as mean ± *SD*.

*ΔΨm* mitochondrial membrane potential, *ATP* adenosine triphosphate, *HCO*_*3*_ bicarbonate, *LDH* lactate dehydrogenase, *MPV* mean platelet volume, *pCO*_*2*_ partial carbon dioxidepressure, *PDW* platelet distribution width, *pO*_*2*_ partial oxygen pressure.

ANOVA with Duncan’s test.

* significantly different values between day 3 or 5 of storage compared to day 1 (*p* < 0.05). ** *p* < 0.001

ATP levels and pH decreased over the storage period. Higher pO_2_ levels were detected on day 5 compared with days 1 and 3, whereas a significant reduction of pCO_2_ levels was detected as storage time increased. Glucose concentration gradually decreased whereas lactate levels increased. There was no significant difference in platelet aggregation when ADP and collagen were used (data not show). However, platelet aggregation increased between days 1 and 5 when arachidonic acid was used (*p* = 0.0082). Percentage of platelets positive for CD61 and CD62P remained stable during storage.

**Fig. 2:**
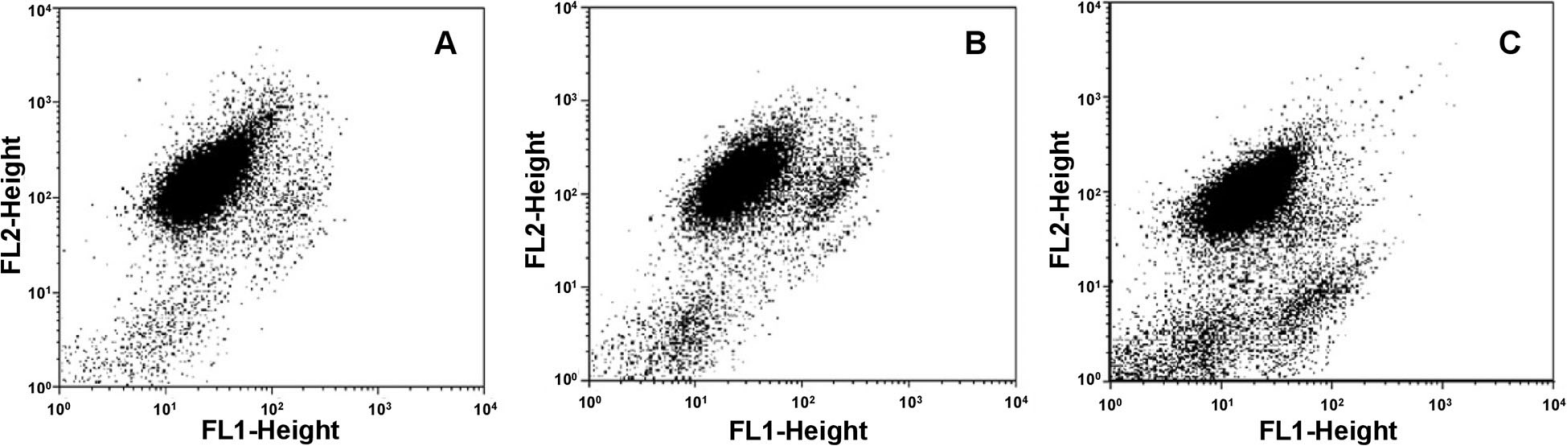
Decrease in mitochondrial membrane potential (∆Ψm) during storage featured by displacement of the cell population from red fluorescence (FL2) to green fluorescence (FL1). An increase on unmarked platelets in the lower left quadrant is observed, suggesting the presence of platelet micro particles **a**) Day 1; **b**) Day 3; **c**) Day 5

Mitochondrial membrane potential (Δψm – JC-1 aggregates) values remained stable between days 1 and 3 of storage. A reduction of Δψm was identified on day 5 when compared with days 1 (*p* = 0.0016) and 3 (*p* = 0.0373) (Fig. 2). No differences in the percentage of cells with phosphatidylserine exposure or with expression of active caspase were observed during the evaluated period. LDH showed a trend to increase (*p* = 0.051).

There was a negative correlation between glucose and lactate (*ρ* = − 0.927, *p* < 0.001), pH and lactate (*ρ* = − 0.871, *p* < 0.001), and lactate and Δψm (ρ = − 0.846, *p* < 0.05). There was a positive correlation between glucose and pH (ρ = 0.936, *p* < 0.001), glucose and swirling (*ρ* = 0.828, *p* < 0.005), glucose and Δψm (*ρ* = 0.911, *p* < 0.001), and pH and swirling (*ρ* = 0.803, *p* < 0.05).

PC were divided into two groups: one with lower than average platelet count (≤ 7.49 × 10^10^/unit, *n* = 14) and one with a higher than average platelet count (> 7.49 × 10^10^/unit, *n* = 8). PC with lower platelet counts presented better quality parameters than those with higher counts on the last day of storage (Table 2).

**Table 2:** Comparison of quality parameters in canine platelet concentrates with high and low platelet counts on day 5 of storage

Parameter	Platelet count (≤ 7.49 × 10^10^/unit) (*n* = 14)	Platelet count (> 7.49 × 10^10^/unit) (*n* = 8)	*P*
PDW (fL)	14.5 ± 4.3	18.4 ± 5.1	0.017
MPV (fL)	12.9 ± 2.6	15.0 ± 4.2	0.043
Glucose (mmol/L)	17.0 ± 4.6	7.9 ± 3.3	< 0.0001
Lactate (mmol/L)	2.1 ± 0.6	4.1 ± 0.9	< 0.0001
ATP (μmol/10^11^platelets)	1.1 ± 0.3	0.8 ± 0.1	< 0.0001
pH (22 °C)	6.3 ± 0.2	5.3 ± 0.4	< 0.0001
pO_2_ (mmHg)	126.6 ± 23.7	152.9 ± 32.6	< 0.0001
pCO_2_ (mmHg)	13.3 ± 3.7	9.1 ± 5.4	0.043
∆Ψm JC-1 (%)	66.5 ± 10.1	15.3 ± 8.7	0.003
LDH (mg/dL)	442.7 ± 76.6	957.7 ± 100.0	0.022

*ΔΨm* mitochondrial membrane potential, *ATP* adenosine triphosphate, *LDH* lactate dehydrogenase, *MPV* mean platelet volume, *pCO*_*2*_ partial carbon dioxidepressure, *PDW* platelet distribution width, *pO*_*2*_ partial oxygen pressure.

## Discussion

In our study, we evaluated stored canine PC on days 1, 3 and 5 of storage with platelet aggregation test, biochemical assays and flow cytometry analysis. Our results were consistent with previous reports from human [12, 13] and canine PC studies [1, 3, 4, 14]. Canine PC showed a decrease in pH, glucose, bicarbonate, swirling and pCO_2_ values, and an increase in lactate and pO_2_ values. Hence, our values for those parameters may be used as references for quality control of canine PC in veterinary blood banks (Table 3).

**Table 3:** Suggested reference values for canine platelet concentrates obtained through platelet-rich plasma method and stored for 5 days in TOTM-plasticized bags

Parameter	Expected value
Volume	50–70 mL
Platelet count ^a^	≤ 7.49 × 10^10^/unit
Residual leukocytes (× 10^8^/unit)	< 2.0
pH	6.59–7.35
Swirling	≥ 2+
Microbiological analisys	Negative

^a^ Platelet concentrate bags stored in TOTM-plasticized bags with platelet counts > 7.49 × 10^10^/unit should be used in less than five days

When PC with lower and higher than average platelet counts were compared, those with higher counts presented significantly lower pH, glucose, ATP and ∆ψm values, and higher lactate concentrations. All PC bags with lower than average platelet counts presented good quality until day 5 of storage, in agreement with recommendations of the Brazilian Health Surveillance Agency (ANVISA) [11]. Similar findings were reported in human PC, with the report of significant differences in glucose, lactate, pH, pCO_2_, bicarbonate and ATP values in PC with high platelet counts on day 5 of storage [15]. Another study also reported a faster decrease of swirling, pH and glucose values in PC with high platelet counts when compared with those with lower platelet counts [16]. Our data on PDW and MPV indicate that platelets in the PC with lower than average counts may present less swelling and lower fragmentation rates. When PC contains a high number of platelets per volume, oxygen is consumed faster, and the metabolic pathway used by platelets becomes anaerobic with consequent lactate production and decrease in pH [15, 16]. Still, a higher plasma volume per bag increases the transfusion volume and the risk of transfusion reactions [7, 9].

The correlations among the studied parameters determined on day 5 are consistent with the alterations suffered by platelets during storage. A significant correlation between pH decrease and increase in MPV and PDW indexes was observed. MPV increase was also correlated with swirling loss, indicating that platelets shape changed from discoid to spherical. The higher lactate concentration on day 5 of storage was correlated with the increase in MPV and strongly correlated with the decreases in swirling and pH. These correlations were expected due to the cause-effect relationship between medium acidity and morphological changes in platelets [17].

The high pO_2_ values by the end of storage period indicates that glycolytic anaerobic metabolism superseded aerobic metabolism despite the presence of oxygen (Warburg effect) [15], as confirmed by lactate accumulation and pH reduction. When platelets divert their metabolism to anaerobic glycolysis, there is a decrease in pH and swirling loss. The positive correlation between swirling loss and ΔΨm decrease may explain the lower oxygen utilization, because the tricarboxylic acid cycle occurs in the mitochondria. Along with pH decrease, bicarbonate values gradually and significantly decreased, because it is mobilized to buffer the accumulated H^+^ ions [5]. As glucose is consumed, lactate levels increase, exceeding the medium buffering capacity and causing a decrease in pH followed by swirling loss [15].

Platelet aggregation capacity increased in response to arachidonic acid, whereas no differences were observed in response to ADP or collagen. However, light transmission aggregometry studies in the clinical practice require fresh platelets, with less than 4 h after blood sampling [18]. This is a limitation of the aggregation tests performed and could explain our results.

The percentage of CD61 expression remained stable during storage, indicating that there was no glycoprotein internalization by endocytosis [19]. P-selectin expression on canine platelets surface did not change over time, in contrary to previous studies with human platelets. However, PC acquisition method may influence platelet activation, since human studies have shown that buffy-coat or apheresis methodologies may increase platelet activation [13, 20] and our samples were obtained with PRP method.

Some authors suggest that ΔΨm loss is the most sensitive marker of platelet quality during storage, because it signals the apoptosis process, even before caspase pathway activation or phosphatidylserine exposure [20–23]. We did not observe an increase in phosphatidylserine exposure, probably due to the short evaluation time [24]. Nonetheless, a reduction in ∆Ψm values was observed from day 3 of storage. Correlations were observed among ΔΨm and classical PC quality markers, such as glucose, lactate and pH, as well as a high correlation between swirling loss and ΔΨm reduction. These results suggest that swirling evaluation may be routinely used as a predictor of PC quality in veterinary blood banks, as it is an efficient and low-cost technique, requiring only a trained evaluator.

In the clinical setting, high platelet concentrations are desirable to achieve higher transfusion efficiency. Nevertheless, considering that PC with higher platelet counts experience more severe PSL during storage, it is suggested that canine PC stored in TOTM-plasticized bags with counts greater than 7.49 × 10^10^ per unit should be used in less than five days, as exceeding this period may negatively affect PC quality and possibly transfusion efficacy.

Our study has some limitations. Samples were not evaluated daily and caspase should have been assessed for longer than 5 days of storage.

## Conclusion

In conclusion, quality parameters of canine platelets under standard storage conditions were similar to those observed in human platelets. Moreover, as the results of the present study are specific of canine PC, they could be used for quality control in veterinary blood banks.

## Methods

### Animal selection

Twenty-two dogs aged between 2 and 7-years old, weighing among 30 kg and 60 kg, with no history of receiving transfusion and not taking any medication were selected. The animals were privately owned. Dogs were individually submitted to physical examination, complete blood counts (CBC), blood chemistry profile, and infectious disease screening (Snap 4Dx Test, Idexx Laboratories Inc., Westbrook, USA; Vetcheck, Tecsa Lab, São Paulo, BRA). Included breeds were Golden Retriever (*n* = 12), German Shepherd (*n* = 4), Giant Schnauzer (*n* = 4) and English Mastiff (*n* = 2). All included dogs had updated vaccines and deworming and were considered healthy.

### Blood collection

After clinical and laboratory approval, a total of 450 mL of whole blood (WB) were collected in bags using a triple-bag closed system (CPD/SAGM, JP Indústria Farmacêutica, São Paulo, BRA). Primary collection bags had sodium citrate as anticoagulant; and citrate, phosphate and dextrose (CPD) solution as an additive solution for red blood cells. Dogs were kept under gentle physical restraint and with no anesthesia for blood collection. After trichotomy and 70% alcohol asepsis, WB was collected by puncturing the jugular vein [25], using equipment with automatic control of homogenization, flow and donation volume (Biomixer 323, Ljungberg & Kögel AB, Helsingborg, SWE). After blood withdrawn, all included dogs received water and a tasty meal, and remained under observation for at least 30 min; subsequently they were released to their owners.

### Platelet concentrates preparation using the platelet-rich plasma method

WB bags were allowed to stand at 22 °C for one hour after collection. The PRP method was applied to obtain the PC as follows: each WB bag was subjected to light centrifugation (1600 X g) for 6 min at 22 °C (Sorvall Legend RT + Centrifuge, Thermo Scientific, Waltham, USA). PRP was removed using a manual plasma extractor (ACS201, Terumo Medical of Brazil, São Paulo, BRA) and then subjected to a second centrifugation (3300 X g) for 8 min at 22 °C. Fluid weight and density (1.026 g/mL) were used to calculate the final volume of the bag [26]. Excessive plasma was removed with the aid of a plasma extractor until 50–70 mL of residual plasma remained at the bottom of the bag with the sedimented platelets [25]. PC were stored in polyvinyl chloride (PVC) bags plasticized with tri-2-ethyl-trimellitate (TOTM, JP Indústria Farmacêutica), which are specific for platelet storage and do not have anticoagulants nor additive solutions.

### Storage and sampling of canine platelet concentrates units

PC bags were left undisturbed for one hour after preparation, and then were stored at 20–24 °C in a platelet environmental chamber (model CDCI 03, Indrel, São Paulo, BRA) under constant stirring (AP48L linear plate agitator, Presvac, BA, ARG). Aliquots were removed for analyses using a sampling site coupler previously sterilized with 70% alchool (Fenwal, Lake Zurich, USA) on days 1, 3, and 5 [4]. All PC were kept out of store for sampling for a maximum of 60 s. On day 5 of storage, all 22 PC were submitted to aerobic and anaerobic microbiological culture in brain heart infusion (BHI) incubated at 37 °C for 10 days.

### Qualitative variables

Immediately before aliquot sampling, platelet swirling was assessed. This technique consists in the visual evaluation of the PC against a light source to observe platelet movement. Swirling is classified according to a 0–3 scale, where 0 indicates no platelet swirling and 3 corresponds to platelets with very nice cloudy movements [27, 28]. Swirling was assessed in all PC bags by the same evaluator. Platelet count, PDW and MPV were determined using an automated hematology counter calibrated for canines (PocH-100iV Diff, Sysmex, Lincolnshire, USA). Residual leukocytes were counted in a Nageotte chamber (LO-Laboroptik GmbH, Bad-Homburg, DEU) on day 1 of storage (24 h after collection), as previously described [15].

### Biochemical analyses

Lactate and LDH concentrations were determined by dry chemistry (Vitros 250 Chemistry System, Jonhson & Jonhson, São Paulo, BRA). Glucose, HCO_3_, pO_2_ and pCO_2_ values were determined using a portable blood gas analyzer (CG8, i-Stat, Abbott Point of Care, Mississauga, CAN), according to the manufacturer’s instructions. On days 1, 3, and 5, ATP was extracted [29] and samples were frozen at − 80 °C until further analyses. Platelet ATP levels were determined by bioluminescence (Lite Luminescence ATP Detection Assay System, PerkinElmer, Waltham, USA), and samples were read in a multi-mode microplate reader (Spectramax M5, Molecular Devices Inc., San Jose, USA) in the same assay. The pH was measured using a calibrated pH meter (HI 99171, Hanna Instruments Inc., Woonsocket, USA) under controlled temperature (22 °C) and according to the manufacturer’s instructions. All samples were tested in duplicate.

### Flow cytometry analyses

Platelet activation was determined according to CD62P (P-selectin) expression. About 500,000 platelets were incubated with titrated amounts of anti-human CD61 FITC (clone VI-PL2, BD Biosciences, San Jose, USA) and anti-human CD62P PE (clone AC1.2, BD Biosciences, San Jose, USA) monoclonal antibodies for 20 min at room temperature. Samples were resuspended in phosphate buffered saline (PBS) prior to acquisition. Percentages of platelets positive for CD61 and CD62P were recorded.

Assessment of Δψm was performed as described elsewhere [30]. Briefly, platelets were incubated with MitoScreen JC-1 (BD Biosciences, San Jose, USA). Cells were pelleted by centrifugation and resuspended in MitoScreen buffer for acquisition. Relative degrees of mitochondrial polarization were quantified by measuring the red-shifted JC-1 aggregates, which are favored under conditions of high membrane potential, and green-shifted monomers, which tend to predominate under conditions of low membrane potential [31].

Phosphatidylserine exposure was assessed with Annexin V. About 500,000 platelets were incubated with ApoFlowEx® FITC Kit (Exbio, Praha, CZE) for 15 min at room temperature, according to the manufacturer’s instructions. Samples were washed once and resuspended in PBS before acquisition. The percentage of platelets binding to Annexin V was recorded.

CaspACE™ FITC-VAD-FMK (Promega, Madison, USA) was used to determine caspases involvement in apoptosis induction. About 5,000,000 platelets were incubated with 1 mM of CaspACE™ FITC-VAD-FMK for 20 min at 37 °C. Samples were washed once and resuspended in 1 mL of PBS before acquisition. Percentage of platelets presenting active caspase was recorded.

All flow cytometry experiments were performed on a BD FACSCalibur flow cytometer (BD Biosciences, San Jose, USA) using CellQuest™ Pro software, after daily quality control procedures. At least 30,000 events were acquired for each analysis. All flow cytometry data were analyzed using FCS Express 5 software (De Novo, Software, Ontario, CAN).

### Platelet aggregation test

Platelet aggregation test was performed with light transmission aggregometry (Agreg Myr4 aggregometer, Qualiterm, Cesário Lange, BRA) using ADP (5 μM), collagen (5 μg/mL), and arachidonic acid (1 μM) as inducers.

### Statistics

Data analysis was carried out with ANOVA for repeated measurements, and means were compared with Duncan’s test. Correlations among the evaluated parameters were tested with Pearson’s correlation test. Results were considered significant at *p* < 0.05. All data were analyzed with GraphPad Prism 6.0 Software (GraphPad Software, La Jolla, USA).

### Ethical aspects

This study was approved by the Ethics Committee on the Use of Animals of the Universidade Federal do Rio Grande do Sul (CEUA/UFRGS) under protocol #26102. The owners of all evaluated dogs provided written informed consent before inclusion in the study.

## Data Availability

The datasets used and/or analyzed during the current study are available from the corresponding author upon reasonable request.

## Abbreviations

ANVISA: Brazilian Health Surveillance Agency
CBC: Complete blood counts
PC: Platelet concentrate
PRP: Platelet-rich plasma
PSL: Platelet storage lesions
PVC: Polyvinyl chloride
WB: Whole blood
Δψm: Mitochondrial membrane potential

## References

[1] Allyson K, Abrams-Ogg AC, Johnstone IB (1997). Room temperature storage and cryopreservation of canine platelet concentrates. Am J Vet Res.

[2] Appleman EH, Sachais BS, Patel R, Drobatz KJ, Groman RP, Kennedy DR (2009). Cryopreservation of canine platelets. J Vet Intern Med.

[3] Klein A, Adamik A, Mischke R (1999). Changes in platelet concentrates from dogs due to storage. I. Platelet count in in vitro function. Berl Munch Tierarztl Wochenschr.

[4] Hlavac N, Lasta CS, Dalmolin ML, Lacerda LA, de Korte D, Marcondes NA (2017). In vitro properties of concentrated canine platelets stored in two additive solutions: a comparative study. BMC Vet Res.

[5] Tynngård N (2009). Preparation, storage and quality control of platelet concentrates. Transfus Apher Sci.

[6] Mittal K, Kaur R (2015). Platelet storage lesion: an update. Asian J Transfus Sci.

[7] Callan MB, Appleman EH, Sachais BS (2009). Canine platelet transfusions. J Vet Emerg Crit Care (San Antonio)..

[8] Davidow EB, Brainard B, Martin LG, Beal MW, Bode A, Ford MJ (2012). Use of fresh platelet concentrate or lyophilized platelets in thrombocytopenic dogs with clinical signs of hemorrhage: a preliminary trial in 37 dogs. J Vet Emerg Crit Care (San Antonio).

[9] Davidow B (2013). Transfusion medicine in small animals. Vet Clin North Am Small Anim Pract.

[10] Holme S, Moroff G, Murphy S (1998). A multi-laboratory evaluation of in vitro platelet assays: the tests for extent of shape change and response to hypotonic shock. Biomedical excellence for safer transfusion working Party of the International Society of blood transfusion. Transfusion.

[11] Boas práticas no ciclo do sangue, RDC N° 34 (2014).

[12] Ghezelbash B, Amini Kafiabad S, Hojjati MT, Hamidpoor M, Vaeli S, Tabtabae MR (2015). In vitro assessment of platelet lesions during 5-day storage in Iranian blood transfusion organization (IBTO) centers. Arch Iran Med.

[13] Jain A, Marwaha N, Sharma RR, Kaur J, Thakur M, Dhawan HK (2015). Serial changes in morphology and biochemical markers in platelet preparations with storage. Asian J Transfus Sci..

[14] Klein A, Adamik A, Mischke R (1999). Changes in platelet concentrates from dogs due to storage. II. Biochemical changes in concentrate plasma. Berl Munch Tierarztl Wochenschr.

[15] Gulliksson H, Sandgren P, Sjodin A, Hultenby K (2012). Storage of platelets: effects associated with high platelet content in platelet storage containers. Blood Transfus.

[16] Feys HB, Devloo R, Sabot B, De Pourcq K, Coene J, Compernolle V (2017). High platelet content can increase storage lesion rates following Intercept pathogen inactivation primarily in platelet concentrates prepared by apheresis. Vox Sang.

[17] Singh H, Chaudhary R, Ray V (2003). Platelet indices as quality markers of platelet concentrates during storage. Clin Lab Haematol.

[18] Cattaneo M., Cerletti C., Harrison P., Hayward C. P. M., Kenny D., Nugent D., Nurden P., Rao A. K., Schmaier A. H., Watson S. P., Lussana F., Pugliano M. T., Michelson A. D. (2013). Recommendations for the standardization of light transmission aggregometry: a consensus of the working party from the platelet physiology subcommittee of SSC/ISTH. Journal of Thrombosis and Haemostasis.

[19] Wilkerson MJ, Shuman W (2001). Alterations in normal canine platelets during storage in EDTA anticoagulated blood. Vet Clin Pathol.

[20] Albanyan AM, Harrison P, Murphy MF (2009). Markers of platelet activation and apoptosis during storage of apheresis- and buffy coat-derived platelet concentrates for 7 days. Transfusion.

[21] Verhoeven AJ, Verhaar R, Gouwerok EG, de Korte D (2005). The mitochondrial membrane potential in human platelets: a sensitive parameter for platelet quality. Transfusion.

[22] Leaver HA, Schou AC, Rizzo MT, Prowse CV (2006). Calcium-sensitive mitochondrial membrane potential in human platelets and intrinsic signals of cell death. Platelets.

[23] Cookson P, Sutherland J, Turner C, Bashir S, Wiltshire M, Hancock V (2010). Platelet apoptosis and activation in platelet concentrates stored for up to 12 days in plasma or additive solution. Transfus Med.

[24] Dasgupta SK, Argaiz ER, Mercado JE, Maul HO, Garza J, Enriquez AB (2010). Platelet senescence and phosphatidylserine exposure. Transfusion.

[25] ABRAMS-OGG ACG, SCHNEIDER A, WEISS DJ, WARDROP KJ (2010). Principles of canine and feline blood collection processing and storage. Schalm's: veterinary hematology. Transfusion medicine.

[26] Van Der Meer PF, Kerkhoffs JL, Curvers J, Scharenberg J, De Korte D, Brand A (2010). In vitro comparison of platelet storage in plasma and in four platelet additive solutions, and the effect of pathogen reduction: a proposal for an in vitro rating system. Vox Sang.

[27] Mathai J, Resmi KR, Sulochana PV, Sathyabhama S, Baby Saritha G, Krishnan LK (2006). Suitability of measurement of swirling as a marker of platelet shape change in concentrates stored for transfusion. Platelets.

[28] Naghadeh HT, Badlou BA, Ferizhandy AS, Mohammadreza TS, Shahram V (2013). Six hours of resting platelet concentrates stored at 22-24 masculineC for 48 hours in permeable bags preserved pH, swirling and lactate dehydrogenase better and caused less platelet activation. Blood Transfus.

[29] Johnson L, Loh YS, Kwok M, Marks DC (2013). In vitro assessment of buffy-coat derived platelet components suspended in SSP+ treated with the INTERCEPT blood system. Transfus Med.

[30] Marcondes Natália Aydos, Terra Silvia Resende, Lasta Camila Serina, Hlavac Nicole Regina Capacchi, Dalmolin Magnus Larruscaim, Lacerda Luciana de Almeida, Faulhaber Gustavo Adolpho Moreira, González Félix Hilário Díaz (2018). Comparison of JC‐1 and MitoTracker probes for mitochondrial viability assessment in stored canine platelet concentrates: A flow cytometry study. Cytometry Part A.

[31] Cossarizza A, Baccarani-Contri M, Kalashnikova G, Franceschi C (1993). A new method for the cytofluorimetric analysis of mitochondrial membrane potential using the J-aggregate forming lipophilic cation 5,5′,6,6′-tetrachloro-1,1′,3,3′-tetraethylbenzimidazolcarbocyanine iodide (JC-1). Biochem Biophys Res Commun.

